# Supplementary material for: Western blot using Trypanosoma cruzi chimeric recombinant proteins for the serodiagnosis of chronic Chagas disease: A proof-of-concept study
Source: PLoS Negl Trop Dis. 2022 Nov 28;16(11):e0010944. doi: 10.1371/journal.pntd.0010944 (PMC9731424; doi:10.1371/journal.pntd.0010944)
Supplement: Attachment — Submitted filename: Reply_PNTD_D_22_01124.pdf [file pntd.0010944.s004.pdf]

Ministério da Saúde

FIOCRUZ
Fundação Oswaldo Cruz
Instituto Gonçalo Moniz

Salvador-Bahia, Brazil - October 2022

Dear Drs. Andrea Angheben and Charles Jaffe,
Editors of PLOS Neglected Tropical Diseases.

We thank you and the reviewers for your thoughtful suggestions that helped improve our manuscript. We believe this revised manuscript is a better and more balanced representation of our research, and we hope it is now suitable for publication in your journal. The answers to the questions can be found below (PNTD-D-22-01124).

Sincerely,

Dr. Fred Luciano Neves Santos
Corresponding author
fred.santos@fiocruz.br

Rua Waldemar Falcão, nº 121, Candeal, Salvador, Bahia, CEP 40296-710, Brasil
Tels. +55 71 3176-2322; +55 71 99390-3004
E-mail: fred.santos@fiocruz.br

Editor's Reply to the Review Report

In addition to observations of the three reviewers, we ask you to pay attention to the following points during revision process:

Question 1. Probably you are not aware about the commercially available (at least in Europe) LDBio western blot for Chagas disease; reviewer 2 is giving you the reference; please revise the paper taking it into account and add the reference.

Reply: Thank you for pointing out this important information. We are not aware that this (and others!) commercial kit exists. We have rewritten some parts of the paper to include this important kit:

Abstract

Before: "PCR offers low sensitivity in the chronic phase, whereas only one confirmatory test based on the WB method is commercially available worldwide."
After (line 30-32): PCR offers low sensitivity in the chronic phase, whereas few confirmatory tests based on the WB method are commercially available worldwide.

Author Summary

Before: "However, only one confirmatory test based on the WB method is commercially available worldwide."
After (line 58-59): However, few confirmatory tests based on the WB method are commercially available worldwide.

Introduction

Before: "…and Western blot assays employing trypomastigote excreted-secreted antigens (TESA-blot). RIPA, a highly complex technique, is labor-intensive, expensive and involves radioactivity, which limits its use [24,25]."
After (line 126-133): …and Western blot assays employing trypomastigote excreted-secreted antigens (TESA-blot; bioMérieux, Brazil), native trypomastigote and amastigote antigens from the CL Brener *T. cruzi* strain (Chagas Western Blot IgG assay - Chagas blot; LDBio Diagnostics, Lyon, France) or a set of *T. cruzi* recombinant antigens: CRA, FRA, TcD, MAP, SAPA, Ag39 and Tc24 (HBK 740 Immunoblot Linhas anti-*T. cruzi*, EMBRABIO, São Paulo-SP, Brazil) and FP10, FP6, FP3 and TcF (Abbott ESA Chagas, Abbott Laboratories, IL, USA). RIPA, a highly complex technique, is labor-intensive, expensive and involves radioactivity, which limits its use [24,25].

Before: "Until 2017, TESA-blot was routinely used for confirmatory testing [30]; however, commercial production has since been discontinued, leaving a gap in the performance of confirmatory CD diagnosis."
After (line 138-143): Until 2015 and 2017, respectively, HBK 740 Immunoblot Linhas anti-*T. cruzi* and TESA-blot were routinely used for confirmatory testing [32]; however, commercial productions have since been discontinued, leaving a gap in the performance of confirmatory chronic CD diagnosis. However, two other WB are commercially available worldwide. The Chagas blot and Abbott ESA Chagas, whose use is limited to Europe and the USA, respectively.

Discussion

Before: No text.
After (line 368-376): Similar to the TESAcruzi, the Chagas Western blot IgG assay (Chagas blot) uses a complex mixture of antigenic molecules to coat a nylon strip. This assay is manufactured by a French company (LDBio Diagnostics) and uses native trypomastigote and amastigote antigens from the CL Brener *T. cruzi* strain (TcIV). In 2021, a study using samples from *T. cruzi*-positive individuals living in endemic areas of Argentina [49] showed that this kit has great potential for use as a confirmatory test (sensitivity and specificity of 100%). However, the authors note that these results must be confirmed in large tests with many sera from different regions of South America before this immunoblot can be considered a universal confirmatory test.

Before: "However, to date no studies have evaluated the use of chimeric proteins as an antigenic matrix in a WB-based diagnostic immunoassay platform. The diagnostic performance obtained here using WB corroborates our previous findings obtained using a variety of diagnostic platforms [16,17,54,18–22,51–53]. Indeed, all four chimeric proteins achieved 100% sensitivity and specificity, with the exception of IBMP-8.3 (95% sensitivity)."
After (line 385-397): However, to date few studies have evaluated the use of chimeric proteins as an antigenic matrix in a WB-based diagnostic immunoassay platform. In 2010, the Abbott ESA Chagas, an immunoblot based on 4 chimeric recombinant *T. cruzi* antigens (FP10, FP6, FP3, and TcF) [52], was evaluated using samples from different groups of *T. cruzi* infected and uninfected individuals. The high clinical and analytical sensitivity values as well as the simplicity of the method led the authors to conclude that the Abbott ESA Chagas could replace RIPA as the confirmatory test of choice for the detection of antibodies to *T. cruzi* [54]. In November 2011, the Abbott ESA Chagas was approved by the US FDA for the confirmation of blood donors who are repeatedly reactive in Chagas screening tests. The diagnostic performance obtained here using WB corroborates our previous findings obtained using a variety of diagnostic platforms [16–22,55–58]. Indeed, all four chimeric proteins achieved 100% sensitivity and specificity, with the exception of IBMP-8.3 (95% sensitivity).

To accommodate the editor's suggestions, we have added a new reference to the manuscript:

Reference #49. Brossas JY, Griselda B, Bisio M, Guihenneuc J, Gulin JEN, Jauréguiberry S, et al. Evaluation of the chagas Western blot IgG assay for the diagnosis of Chagas disease. Pathogens. 2021;10. doi:10.3390/pathogens10111455

Rua Waldemar Falcão, nº 121, Candeal, Salvador, Bahia, CEP 40296-710, Brasil
Tels. +55 71 3176-2322; +55 71 99390-3004
E-mail: fred.santos@fiocruz.br

Ministério da Saúde

FIOCRUZ
Fundação Oswaldo Cruz
Instituto Gonçalo Moniz

Question 2. In the introduction and discussion give more notes on the problem of discordance in Chagas disease diagnosis (ref http://link.springer.com/10.1007/s10096-011-1393-9) taking into account the concept of the at least 50% rate of Chagas disease affected individuals among the discordant (see Moure et al DOI: https://doi.org/10.1016/j.cmi.2016.06.001, DOI: 10.1016/j.actatropica.2018.05.010).

Reply: Thank you for pointing out this important information. We have rewritten the text to reflect the editor's suggestions.

Introduction

Before: "Following WHO recommendations, around 5% of samples submitted to dual-serological assay testing return discordant or doubtful results (values falling within the cutoff range or indeterminate zone). While initial testing can be repeated, in some cases a third, preferably confirmatory test has also been used…"
After (line 120-124): Following WHO recommendations, around 5% of samples submitted to dual-serological assay testing return discordant or doubtful results (values falling within the cutoff range or indeterminate zone). Some studies have reported similar values ranging from 2.9% to 3.3% [23,24]. While initial testing can be repeated, in some cases a third, preferably confirmatory test has also been used…

Discussion

Before: "Several researchers have investigated the usefulness of WB in the confirmatory diagnosis of CD when conventional tests returned inconclusive results, reporting high values of sensitivity and specificity [40–43]. In 1986, a study used WB to assess the performance of epimastigote antigens in diagnosing CD; however, the low purity of the antigens employed resulted in cross reactions against anti-Leishmania braziliensis and anti-Leishmania donovani [44]."
After (line 339-347): Several researchers have investigated the usefulness of WB in the confirmatory diagnosis of chronic CD when conventional tests returned inconclusive results, reporting high values of sensitivity and specificity [42–45]. The development a methodology to clarify results that are inconclusive after using conventional assays is extremely necessary because at least 50% of discordant results are from individuals affected by Chagas disease [23]. In 1986, a study used WB to assess the performance of epimastigote antigens in diagnosing chronic CD; however, the low purity of the antigens employed resulted in cross reactions against anti-Leishmania braziliensis and anti-*Leishmania donovani* [46].

To accommodate the editor's suggestions, we have added two new references to the manuscript:

- Reference #23. Moure Z, Angheben A, Molina I, Gobbi F, Espasa M, Anselmi M, et al. Serodiscordance in chronic Chagas disease diagnosis: a real problem in non-endemic countries. Clin Microbiol Infect. 2016;22: 788–792. doi:10.1016/j.cmi.2016.06.001
- Reference # 24. Lapa JS, Saraiva RM, Hasslocher-Moreno AM, Georg I, Souza AS, Xavier SS, et al. Dealing with initial inconclusive serological results for chronic Chagas disease in

Rua Waldemar Falcão, nº 121, Candeal, Salvador, Bahia, CEP 40296-710, Brasil
Tels. +55 71 3176-2322; +55 71 99390-3004
E-mail: fred.santos@fiocruz.br

clinical practice. Eur J Clin Microbiol Infect Dis. 2012;31: 967–974. doi:10.1007/s10096-011-1393-9

Question 3. In the conclusions, clearly state that this is a phase I study and a larger sample study is needed to confirm results.

Reply: We thank the editor for this valuable contribution. We have clearly state that this is a phase I study. The text already state that a new study (phase II) will be performed involving an increased number of samples (please consider reading the underlined text below):

Before: The present study represents the first attempt to assess the use of chimeric recombinant proteins using the WB platform. The results obtained herein demonstrate the high discriminatory capacity of all four IBMP antigens to efficiently and safely differentiate T. cruzi-positive from -negative samples; however, a phase II study involving an increased number of samples will serve to further elucidate and confirm the presently described findings.
After (line 440-445): The present study (phase I) represents the first attempt to assess the use of chimeric recombinant proteins using the WB platform. The results obtained herein demonstrate the high discriminatory capacity of all four IBMP antigens to efficiently and safely differentiate T. cruzi-positive from -negative samples; however, a phase II study involving an increased number of samples will serve to further elucidate and confirm the presently described findings.

Reviewer's Responses to Questions

Question 1. Reviewer #2: The authors say (line 56) : there is no reliable test that could serve as a gold standard. There are published data (pathogens 2021 Nov 10;10(11):1455) about a commercialized CE marketed Western blot for the diagnosis of Chagas disease maybe it would be interesting to add this paper in the references and to take it in charge in the discussion

Reply: Thank you for pointing out this important information. We are not aware that this (and others!) commercial kit exists. We have rewritten some parts of the paper to include this important kit:

Abstract

Before: "PCR offers low sensitivity in the chronic phase, whereas only one confirmatory test based on the WB method is commercially available worldwide."
After (line 30-32): PCR offers low sensitivity in the chronic phase, whereas few confirmatory tests based on the WB method are commercially available worldwide.

Author Summary

Before: "However, only one confirmatory test based on the WB method is commercially available worldwide."
After (line 58-59): However, few confirmatory tests based on the WB method are commercially available worldwide.

Introduction

Before: "…and Western blot assays employing trypomastigote excreted-secreted antigens (TESA-blot). RIPA, a highly complex technique, is labor-intensive, expensive and involves radioactivity, which limits its use [24,25]."
After (line 126-133): …and Western blot assays employing trypomastigote excreted-secreted antigens (TESA-blot; bioMérieux, Brazil), native trypomastigote and amastigote antigens from the CL Brener *T. cruzi* strain (Chagas Western Blot IgG assay - Chagas blot; LDBio Diagnostics, Lyon, France) or a set of *T. cruzi* recombinant antigens: CRA, FRA, TcD, MAP, SAPA, Ag39 and Tc24 (HBK 740 Immunoblot Linhas anti-*T. cruzi*, EMBRABIO, São Paulo-SP, Brazil) and FP10, FP6, FP3 and TcF (Abbott ESA Chagas, Abbott Laboratories, IL, USA). RIPA, a highly complex technique, is labor-intensive, expensive and involves radioactivity, which limits its use [24,25].

Before: "Until 2017, TESA-blot was routinely used for confirmatory testing [30]; however, commercial production has since been discontinued, leaving a gap in the performance of confirmatory CD diagnosis."
After (line 138-143): Until 2015 and 2017, respectively, HBK 740 Immunoblot Linhas anti-*T. cruzi* and TESA-blot were routinely used for confirmatory testing [32]; however, commercial productions have since been discontinued, leaving a gap in the performance of confirmatory chronic CD diagnosis. However, two other WB are commercially available worldwide. The Chagas blot and Abbott ESA Chagas, whose use is limited to Europe and the USA, respectively.

Discussion

Before: No text.
After (line 368-376): Similar to the TESAcruzi, the Chagas Western blot IgG assay (Chagas blot) uses a complex mixture of antigenic molecules to coat a nylon strip. This assay is manufactured by a French company (LDBio Diagnostics) and uses native trypomastigote and amastigote antigens from the CL Brener *T. cruzi* strain (TcIV). In 2021, a study using samples from *T. cruzi*-positive individuals living in endemic areas of Argentina [49] showed that this kit has great potential for use as a confirmatory test (sensitivity and specificity of 100%). However, the authors note that these results must be confirmed in large tests with many sera from different regions of South America before this immunoblot can be considered a universal confirmatory test.

Before: "However, to date no studies have evaluated the use of chimeric proteins as an antigenic matrix in a WB-based diagnostic immunoassay platform. The diagnostic performance obtained here using WB corroborates our previous findings obtained using a variety of diagnostic platforms [16,17,54,18–22,51–53]. Indeed, all four chimeric proteins achieved 100% sensitivity and specificity, with the exception of IBMP-8.3 (95% sensitivity)."
After (line 385-397): However, to date few studies have evaluated the use of chimeric proteins as an antigenic matrix in a WB-based diagnostic immunoassay platform. In 2010, the Abbott ESA Chagas, an immunoblot based on 4 chimeric recombinant *T. cruzi* antigens (FP10, FP6, FP3, and TcF) [52], was evaluated using samples from different groups of *T. cruzi* infected and uninfected individuals. The high clinical and analytical sensitivity values as well as the simplicity of the method led the

Rua Waldemar Falcão, nº 121, Candeal, Salvador, Bahia, CEP 40296-710, Brasil
Tels. +55 71 3176-2322; +55 71 99390-3004
E-mail: fred.santos@fiocruz.br

authors to conclude that the Abbott ESA Chagas could replace RIPA as the confirmatory test of choice for the detection of antibodies to *T. cruzi* [54]. In November 2011, the Abbott ESA Chagas was approved by the US FDA for the confirmation of blood donors who are repeatedly reactive in Chagas screening tests. The diagnostic performance obtained here using WB corroborates our previous findings obtained using a variety of diagnostic platforms [16–22,55–58]. Indeed, all four chimeric proteins achieved 100% sensitivity and specificity, with the exception of IBMP-8.3 (95% sensitivity).

To accommodate the editor's suggestions, we have added a new reference to the manuscript:

Reference #49. Brossas JY, Griselda B, Bisio M, Guihenneuc J, Gulin JEN, Jauréguiberry S, et al. Evaluation of the chagas Western blot IgG assay for the diagnosis of Chagas disease. Pathogens. 2021;10. doi:10.3390/pathogens10111455

Reviewer Comments (the reviewer is not known to the authors)

Question 1. Reviewer not identified. Background. Line 134:"…performance of confirmatory CD diagnosis" should be "...performance of confirmatory chronic CD diagnosis"

Reply: Thank you for pointing out this important information. We have changed the text according to the reviewer's suggestion.

Before: "…however, commercial production has since been discontinued, leaving a gap in the performance of confirmatory CD diagnosis."
After (line 139-141): …however, commercial productions have since been discontinued, leaving a gap in the performance of confirmatory chronic CD diagnosis.

Question 2. Reviewer not identified. Background. Line 163: typo error "Laemmili buffer" à "Laemmli buffer".

Reply: We thank the reviewer for this comment and have changed the text according to the reviewer's suggestion.

Before: "…Purified IBMP proteins were individually dissolved in Laemmili buffer and separated…"
After (line 169): "…Purified IBMP proteins were individually dissolved in Laemmli buffer and separated…"

Question 3. Reviewer not identified. Background. Line 166: pore size of nitrocellulose membrane = 0.2 um or 0.45 um?

Reply: We thank the reviewer for calling this to our attention. Pore size of nitrocellulose membrane = 0.45 µm. We have included this information in the text as follows:

Before: "Proteins were transferred to nitrocellulose membrane…"
After (line 171-172): "…Proteins were transferred to a 0.45 µm pore-size nitrocellulose membrane…"

Question 4. Reviewer not identified. Background. Line 169: Please describe the TBS buffer abbreviation.

Reply: We thank the reviewer for this comment. We have inserted the abbreviation in the text as follows:

Before: "Following blotting, the membrane was blocked in TBS containing 0.05% (v/v)…"
After (line 175-176): "Following blotting, the membrane was blocked in Tris-buffered saline (TBS) containing 0.05% (v/v)…"

Question 5. Reviewer not identified. Results. Line 262: 12.5 ng of each IBMP per strip. As the width of strips varies, it is better to inform the reader of the strip's width. For example, 12.5 ng/4 mm!

Reply: We thank the reviewer for this comment. We used a miniblotter system with a constant width of the strips: 4 mm. For more details (if needed), we kindly ask the reviewer to read the data sheet available at https://www.interchim.fr/ft/B/BA369c.pdf. To clarify the width of the strips, we have added a new explanation to the Western Blot Assay subsection in the Materials and Methods section, which reads as follows.

Material and Methods

Before: No text.
After (line 176-178): The membrane was placed on a miniblotter (Miniblotter 25, Immunetics, USA) with 25 channels (4.0 mm each).

Discussion

Before: "…a smaller amount of antigen was used: 12.5 ng for each of the four…"
After (line 416): …a smaller amount of antigen was used: 12.5 ng/4 mm for each of the four…

Question 6. Reviewer not identified. Discussion. Line 332, 335, 339, 349, 401: Please correct the error similar to line 134 (see above). The authors used the correct term in the following lines 353, 356.

Reply: Thank you for pointing out this important information. We have changed the text according to the reviewer's suggestion.

Before: "…WB in the confirmatory diagnosis of CD..."
After (line 340): …WB in the confirmatory diagnosis of chronic CD...

Before: "…antigens in diagnosing CD..."

Rua Waldemar Falcão, nº 121, Candeal, Salvador, Bahia, CEP 40296-710, Brasil
Tels. +55 71 3176-2322; +55 71 99390-3004
E-mail: fred.santos@fiocruz.br

After (line 345-346): …antigens in diagnosing chronic CD...

Before: "…laboratory-based CD diagnosis..."
After (line 349-350): …laboratory-based chronic CD diagnosis...

Before: "…involving the diagnosis of CD via WB..."
After (line 360): …involving the diagnosis of chronic CD via WB...

Before: "…conventional serological diagnosis of CD ..."
After (line 428): …conventional serological diagnosis of chronic CD ...

Question 7. Reviewer not identified. Discussion. Line 386 and 387 – the amount of antigen information is not making sense without information on the strip. It is per well (of x mm width), per mm of strip width, etc. Please make clear about this information!

Reply: We thank the reviewer for this comment. We used a miniblotter system with a constant width of the strips: 4 mm. For more details (if needed), we kindly ask the reviewer to read the data sheet available at https://www.interchim.fr/ft/B/BA369c.pdf. To clarify the width of the strips, we have added a new explanation to the Western Blot Assay subsection in the Materials and Methods section, which reads as follows.

Material and Methods

Before: No text.
After (line 176-178): The membrane was placed on a miniblotter (Miniblotter 25, Immunetics, USA) with 25 channels (4.0 mm each).

Discussion

Before: "…a smaller amount of antigen was used: 12.5 ng for each of the four…"
After (line 416): …a smaller amount of antigen was used: 12.5 ng/4 mm for each of the four…

Conclusion

The study was carried out nicely, with no major concerns. The main concern about this study is that the authors wrote the study results as a phase I study while the authors in 2019 (lines 402-404) evaluated the four IBMP antigens using 600 cutaneous and 229 visceral leishmaniasis samples. These IBMP antigens have been evaluated extensively by authors in ELISA format, so using these antigens in western blot format should be reported as a full report and not as a proof-of-concept or phase 1 study. In a different issue, the study used the best platform of Western Blot using chemiluminescent substrate and using the best reader. It is interesting to test the performance of the assay in different laboratories using the most available substrate for the Western Blot as the goal of this study is to fill the gap on the lack of confirmatory commercial chronic CD diagnosis.

Reply: We thank the reviewer for this valuable contribution. We have clearly state that this is a phase I study. The text already state that a new study (phase II) will be performed involving an increased number of samples (please consider reading the underlined text below):

Before: The present study represents the first attempt to assess the use of chimeric recombinant proteins using the WB platform. The results obtained herein demonstrate the high discriminatory capacity of all four IBMP antigens to efficiently and safely differentiate T. cruzi-positive from -negative samples; however, a phase II study involving an increased number of samples will serve to further elucidate and confirm the presently described findings.
After (line 440-445): The present study (phase I) represents the first attempt to assess the use of chimeric recombinant proteins using the WB platform. The results obtained herein demonstrate the high discriminatory capacity of all four IBMP antigens to efficiently and safely differentiate T. cruzi-positive from -negative samples; however, a phase II study involving an increased number of samples will serve to further elucidate and confirm the presently described findings.
