# Supplementary material for: Western blot using Trypanosoma cruzi chimeric recombinant proteins for the serodiagnosis of chronic Chagas disease: A proof-of-concept study
Source: PLoS Negl Trop Dis. 2022 Nov 28;16(11):e0010944. doi: 10.1371/journal.pntd.0010944 (PMC9731424; doi:10.1371/journal.pntd.0010944)
Supplement: Attachment — Submitted filename: Reviewer Comments on chimeric T cruzi PlosNTD 14SEP2022.docx [file pntd.0010944.s003.docx]

**Reviewer Comments**

**Paper Summary**

               The manuscript reported the development of a Western blot (WB) assay using Trypanosoma cruzi chimeric recombinant proteins for the serodiagnosis of chronic Chagas disease as a proof-of-concept (phase I) study.

**Strength of the Paper**

               The four chimeric recombinant proteins had been used to develop successful assays previously, and the authors investigated these antigens for developing a confirmatory test for chronic Chagas disease. The effort was to fill the gap of no WB-based confirmatory test commercially available since 2017.

**Major weakness**

None

**Minor Comment**

**Background**

Line 134:"…performance of confirmatory CD diagnosis" should be "..performance of confirmatory chronic CD diagnosis"

Line 163: typo error “Laemmili buffer” à “Laemmli buffer”

Line 166: pore size of nitrocellulose membrane = 0.2 um or 0.45 um?

Line 169: Please describe the TBS buffer abbreviation

**Results**

Line 262: 12.5 ng of each IBMP per strip. As the width of strips varies, it is better to inform the reader of the strip's width. For example, 12.5 ng/4 mm!

**Discussion**

Line 332, 335, 339, 349, 401: Please correct the error similar to line 134 (see above). The authors used the correct term in the following lines 353, 356.

Line 386 and 387 – the amount of antigen information is not making sense without information on the strip. It is per well (of x mm width), per mm of strip width, etc. Please make clear about this information!

**Conclusion**

The study was carried out nicely, with no major concerns.

The main concern about this study is that the authors wrote the study results as a phase I study while the authors in 2019 (lines 402-404) evaluated the four IBMP antigens using 600 cutaneous and 229 visceral leishmaniasis samples. These IBMP antigens have been evaluated extensively by authors in ELISA format, so using these antigens in western blot format should be reported as a full report and not as a proof-of-concept or phase 1 study.

In a different issue, the study used the best platform of Western Blot using chemiluminescent substrate and using the best reader. It is interesting to test the performance of the assay in different laboratories using the most available substrate for the Western Blot as the goal of this study is to fill the gap on the lack of confirmatory commercial chronic CD diagnosis.
